# High-fat and high-glucose microenvironment decreases Runx2 and TAZ expression and inhibits bone regeneration in the mouse

**DOI:** 10.1186/s13018-019-1084-2

**Published:** 2019-02-18

**Authors:** Xuan Wu, Yunpeng Zhang, Yixiao Xing, Bin Zhao, Cong Zhou, Yong Wen, Xin Xu

**Affiliations:** 1 Shandong Provincial Key Laboratory of Oral Tissue Regeneration, School of Stomatology, Shandong University, Jinan, 250012 Shandong People’s Republic of China; 2 School of Stomatology, Shandong University, No. 44-1, Wenhua Xi Road, Jinan, China

**Keywords:** T2DM (type 2 diabetes mellitus), Hyperlipidemia, Bone regeneration, Runx2 (runt-related transcription factor 2), TAZ (transcriptional co-activator with PDZ-binding domain)

## Abstract

**Background:**

Type 2 diabetes mellitus (T2DM) and hyperlipidemia are negatively related to bone regeneration. The aim of this study was to evaluate the effect of high-fat and high-glucose microenvironment on bone regeneration and to detect the expression of runt-related transcription factor 2 (Runx2) and transcriptional co-activator with PDZ-binding domain (TAZ) during this process.

**Methods:**

After establishing a high-fat and high-glucose mouse model, a 1 mm × 1.5 mm bone defect was developed in the mandible. On days 7, 14, and 28 after operation, bone regeneration was evaluated by hematoxylin-eosin staining, Masson staining, TRAP staining, and immunohistochemistry, while Runx2 and TAZ expression were detected by immunohistochemistry, RT-PCR, and Western blot analysis.

**Results:**

Our results showed that the inhibition of bone regeneration in high-fat and high-glucose group was the highest among the four groups. In addition, the expression of Runx2 in high-fat, high-glucose, and high-fat and high-glucose groups was weaker than that in the control group, but the expression of TAZ only showed a decreasing trend in the high-fat and high-glucose group during bone regeneration.

**Conclusions:**

In conclusion, these results suggest that high-fat and high-glucose microenvironment inhibits bone regeneration, which may be related to the inhibition of Runx2 and TAZ expression.

**Electronic supplementary material:**

The online version of this article (10.1186/s13018-019-1084-2) contains supplementary material, which is available to authorized users.

## Introduction

The oral and maxillofacial bone structure is an important basis for supposing the face. However, infection, trauma, long-term chronic inflammation, and tumor are the cause of oral and maxillofacial bone defects [1, 2]. In the oral clinics, mandibular defects are common, but the treatments to promote bone regeneration and reconstruction are inadequate [3, 4]. In the clinics, there is a category of patients who not only have bone defects, but also suffer from type 2 diabetes mellitus (T2DM) and hyperlipidemia concurrently. Diabetes mellitus (DM) and hyperlipidemia (common chronic diseases) are reaching epidemic proportions worldwide, and 90–95% of the diagnosed cases of DM in adults are T2DM [5, 6]. Notably, there is a close relationship between T2DM and hyperlipidemia, and a study on 25,817 Chinese T2DM out-patients in 104 hospitals across the country showed that 42% of T2DM patients had hyperlipidemia [7].

As a chronic systemic disease that affects the bones, T2DM contributes to osteoporosis, increases fracture risk, and delays bone healing [8–11]. However, current studies on the association between T2DM and bone formation have mainly focused on simple high-fat or high-glucose condition. Several studies of bone mass of femur and tibia showed that bone regeneration was decreased while bone resorption was increased in mice with hyperlipidemia [12, 13]. Therefore, it is important to establish animal models that simulate both high-fat and high-glucose microenvironments. And APOE^−/−^ mouse is commonly used as an animal models of hyperlipidemia [14]. Therefore, in this experiment, we plan to build a high-fat and high-glucose mouse model based on AOPE^−/−^ mice using the modeling method of T2DM [14, 15].

Currently, most approaches to bone regeneration focus on two aspects: enhancing the osteogenic differentiation of osteoblasts or decreasing the number and activity of osteoclasts. And increasing the differentiation of osteoblasts is the main direction of current studies, such as direct delivery of factors for osteoblasts such as bone morphogenetic protein 2 (BMP2), osterix (Osx), runt-related transcription factor 2 (Runx2) [16–18]. Runx2 is an important transcription factor that regulates osteoblast differentiation [19–21]. Transcriptional co-activator with PDZ-binding domain (TAZ) is a transcriptional coactivator with a PDZ-binding motif that regulates cell differentiation, proliferation, and development [22, 23]. TAZ stimulates osteoblastic differentiation by interacting with Runx2 and stimulating Runx2-mediated gene transcription [22, 25, 26]. TAZ-Runx2 pathway plays an important role in osteogenic differentiation [4]. In our previous study, we showed that TAZ-regulated bone tissue remodeling through Runx2 during orthodontic tooth movement, and Runx2, and TAZ were co-localized in osteoblasts [27].

Previous studies reported that the expression of Runx2 was suppressed in both high-fat condition and high-glucose conditions [10, 13]. However, the expression of Runx2 and TAZ during the bone regeneration process under high-fat and high-glucose microenvironment has not been investigated. Therefore, in this study we first evaluated the effects of high-fat and high-glucose microenvironment on bone regeneration in mouse model. We further examined the expression of Runx2 and TAZ during bone regeneration to investigate their role in bone regeneration under high-fat and high-glucose microenvironment.

## Materials and methods

### Animals and tissue preparation

A total of 60 6-week-old male wild-type mice (C57BL/6J) and 60 6-week-old male apolipoprotein e knockout mice (APOE^−/−^; on C57 BL/6J background) (weight 16 g on average) were purchased from the Laboratory Animal Center of Shandong University (Jinan, China) and Nanjing Institute of Biomedical Research of Nanjing University (Nanjing, China), respectively. All animal experiments were performed in accordance with the guidelines for the care and use laboratory animals and were approved by the Institutional Animal Care and Use Committee of Shandong University.

Animals were divided to four groups: Group NC (normal control), C57BL/6J mice (*n* = 30), fed on a chow diet; Group HF (high-fat), APOE^−/−^ mice (*n* = 30), fed on a high-fat diet; Group HG (high-glucose), C57BL/6J mice (*n* = 30), fed on a high-fat and high-sucrose diet; and Group HFHG (high-fat and high-glucose), APOE^−/−^ mice (*n* = 30), fed on a high-fat and high-sucrose diet.

**Fig. 1:**
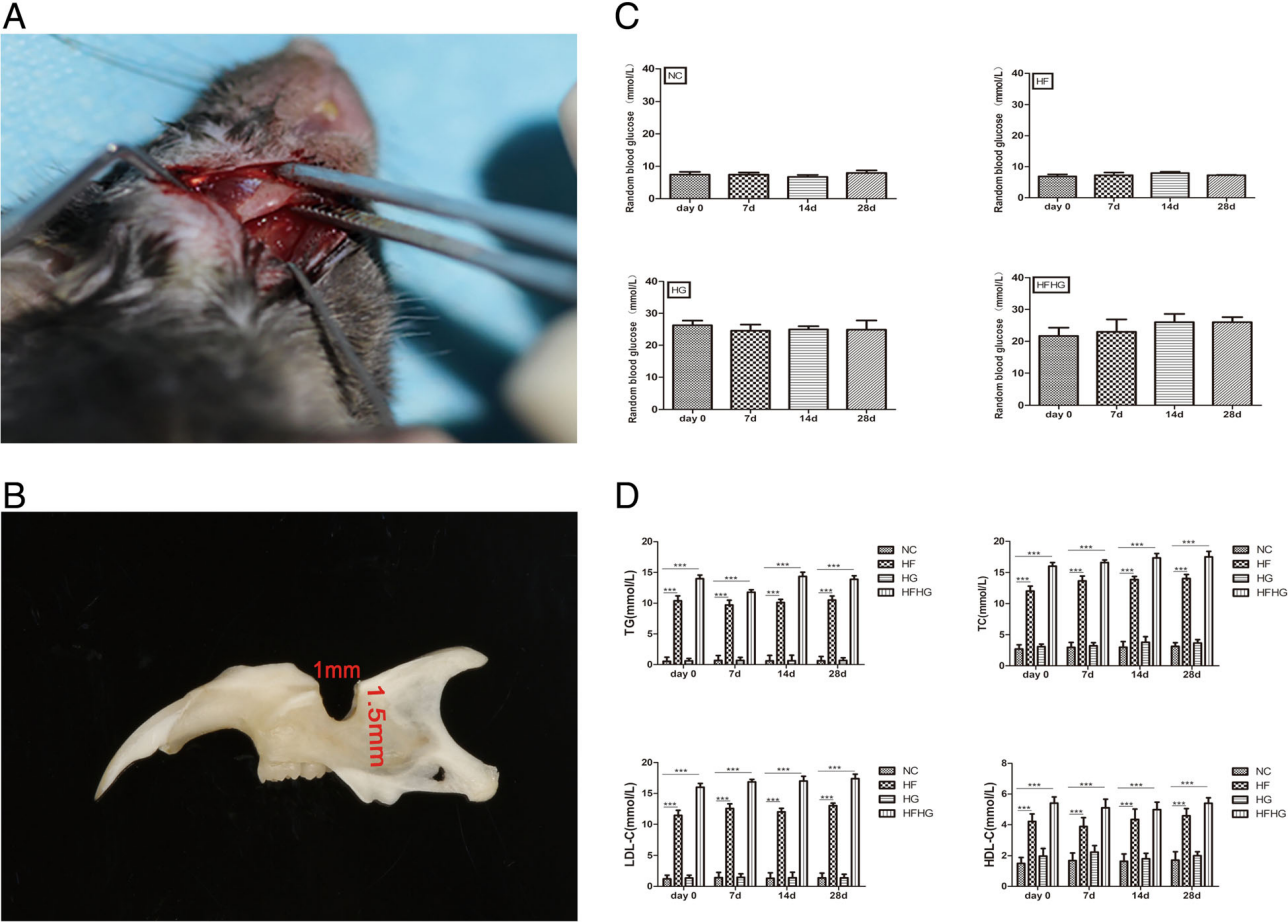
Bone defect model and metabolic parameters. **a** The right mandible of the mouse was incised to completely expose the buccal and lingual side. **b** The mandible was made with a 1 mm × 1.5 mm defect across buccal to lingual. **c** The blood glucose level was above 16.9 mmol/l in HG and HFHG groups. **d** The TC, TG, LDL-C, and HDL-C levels in HF and HFHG groups increased significantly compared with NC and HG group. *p < 0.05, **p < 0.01, ***p < 0.001

After feeding for 4 weeks, T2DM was induced in mice from the HG and HFHG groups via a single intraperitoneal injection of streptozotocin (STZ; Sigma, St Louis, MO, USA) dissolved in fresh sodium citrate buffer (0.1 M, pH 4.2) at a dose of 100 mg/kg body weight. The blood glucose was monitored via blood glucose meter (Sannuo biosensing co., Ltd.; China) at days 0 (before the operation),7 (after the operation), 14 (after the operation), and 28 (after the operation). After successful establishment of T2DM model, blood lipid, including total cholesterol (TC), triglycerides (TG), low-density lipoprotein (LDL-C), and high-density lipoprotein (HDL-C), was detected at the Laboratory of Qilu Hospital (Jinan, China) at days 0, 7, 14, and 28. Then, the same age mice were anesthetized with intraperitoneal injection of sodium pentobarbital (Sigma). The right submandibular incision was used to expose the lower edge of the mandibular body and the side of the buccal tongue (Fig. 1a). A bone defect area of 1 mm × 1.5 mm across the buccal to the lingual was created on the mandible (Fig. 1b).

The right mandibular tissue of the mice was isolated under anesthesia with intraperitoneal injection of chloral hydrate on days 7 (*n* = 10), 14 (*n* = 10), and 28 (*n* = 10). The separated mandible was immersed in 4% PFA at 4 °C and fixed for 24 h. Thereafter, the tissue was decalcified with 10% EDTA for 4 weeks. Then, the tissues were subjected to alcohol gradient dehydration and embedded in paraffin after being made transparent with xylene. Serial longitudinal 5-μm-thick sections were prepared for subsequent histological analysis using a sliding microtome.

The tissue specimen of mandibular bone defects in mice that were for qPCR and Western only comprised of the defect site and an extremely small portion of the normal surrounding bone tissue. In the experiment, tissues were obtained 7, 14, and 28 days after surgery. Then, the tissues were stored in liquid nitrogen.

### Hematoxylin and eosin (HE) staining

Sections were deparaffinized with xylene and rehydrated in a graded alcohol series. After being washed with double distilled water (DDW), sections were stained with hematoxylin, differentiation solution, and eosin in order (Sigma). At last, sections were dehydrated through a graded ethanol series, cleared with xylene, and mounted with neutral resins. Stained sections were observed and digital images were taken with a light microscope, and new bone formation rate (New bone area/bone defect area × 100%) was calculated by Image-Pro Plus software (version 6.2; Media Cybernetics, Rockville, MD, USA). Specifically, six slices of each sample were used for quantitative analysis.

### Modified Masson staining

The sections were deparaffinized in xylene, hydrated through a series of graded alcohol and dewaxed, then sliced into the Bouin liquid (Leagene, Beijing, China) for 2 h mordant at 37 °C and washed with water until the yellow slices disappeared. After staining with celestite blue for 3 min and Mayer’s hematoxylin stain for 3 min, the acidic ethanol differentiation solution was then differentiated for 1 s, rinsed with water for 10 min, and stained with red magenta for 10 min. The slices were then incubated in phosphomolybdic acid solution for 10 min, in aniline blue staining solution for 5 min, and in weak acid solution for 2 min. Finally, the sections were dehydrated by alcohol and made transparent by xylene treatment, and the resin was sealed.

### Immunohistochemistry (IHC) analysis

Briefly, the sections were deparaffinized in xylene, hydrated through a series of graded alcohol, and washed with 0.1 mmol/l PBS. Antigen retrieval was performed by treatment with 0.125% trypsin (Zhongshan, Beijing, China) at 37 °C for 20 min. The activity of endogenous tissue peroxidase was blocked with 3% hydrogen peroxide (Zhongshan) for 30 min. After pretreatment with normal goat serum (Zhongshan) for 30 min to block nonspecific binding, the sections were incubated with antibodies for ALP (1:200, rabbit anti-serum against rat, Abcam, Cambridge, MA, USA), Runx2 (1:250, rabbit anti-serum against rat, Cell Signaling Technology, Danvers, MA, USA), and TAZ (1:400, rabbit anti-serum against rat, Cell Signaling Technology) at 4 °C overnight. The sections were stained by avidin-biotin-peroxidase complex technique, followed by reaction with diaminobenzidine, and counterstaining with hematoxylin. Immunostaining intensities (optical density, OD) were analyzed using Image-Pro Plus 6.2 software. At least six sections from each sample were analyzed.

### Tartrate-resistant acid phosphatase (TRAP) staining

TRAP Kit (Sigma-Aldrich, St. Louis, MO, USA) was used for TRAP staining to evaluate osteoclasts. The sections were deparaffinized in xylene, hydrated through a graded alcohol series, and incubated at 37 °C for 30 min in AS-BI phosphate in acetate-tartrate buffer. Then, the sections were incubated in sodium nitrite solution for 60 min at 37 °C and counterstained with hematoxylin. Six stained sections were observed under an Olympus microscope and the number of TRAP positive cell was analyzed. The number of osteoclasts was counted in three randomly selected non-overlapping microscopic fields in each section by Image-Pro Plus 6.2 software.

### qRT-PCR

Total RNA was extracted using Trizol reagent according to the manufacturer’s protocol (Invitrogen, Frederick, USA). One microgram of RNA was reverse-transcribed to cDNA with PrimeScriptR RT reagent kit (TaKaRa Tokoy, Japan). cDNA (2 μl) was amplified and quantified (Bio-Rad laboratories, Inc., CA, USA). Each sample was analyzed in triplicate. GAPDH was used as an endogenous control. The relative mRNA expression level was normalized to that of GAPDH and analyzed with the 2^−△△CT^ method. The primers sequences are showed in Table 1.

**Table 1:** The sequences of primers

Genes	Primers	Primers sequence (5′-3′)
GAPDH	Forward	GTGGTAGGCAGTCCCACTTT
	Reverse	GAGCACTCACTGACTCGGTT
Runx2	Forward	CTAGCCAGCAGCGTCGTCATG
	Reverse	GCAAGACTGGTGGTTGGAGACG
TAZ	Forward	TGCACCACCAACTGCTTAG
	Reverse	GGATGCAGGGATGATGTTC

### Western blot analysis

The tissues were thoroughly ground in a liquid nitrogen environment and lysed with RIPA (Solarbio, Beijing, China) at 4 °C for 30 min. The tissues were harvested and centrifuged at 4 °C and 12,000 rpm for 30 min. The protein concentration was determined by the BCA assay. Twenty micrograms of protein from each group was separated on 10% SDS-polyacrylamide gels and transferred onto polyvinylidene difluoride membranes (Millipore, Bedford, MA, USA). Membranes were blocked with 5% fat-free milk in Tris-buffered saline containing 0.1% Tween-20 (TBST), then incubated overnight at 4 °C with primary antibodies against TAZ (1:10,000, Cell Signaling Technology, Danvers), Runx2 (1:10,000, Cell Signaling Technology), and GAPDH (1:20,000, Proteintech, Chicago, IN, USA). GAPDH was used as an internal control for normalization. The membranes were washed three times with TBST and incubated with goat anti-mouse or anti-rabbit HRP-conjugated secondary antibodies (1:10,000) for 1 h at room temperature. The bands were detected by chemiluminescence system and analyzed by ImageJ software (National Institutes of Health, Bethesda, MD, USA).

### Statistical analysis

Data were presented as means ± SD and analyzed using SPSS statistical software (SPSS 14.0, Chicago, IL, USA). The difference among the groups was compared by one-way ANOVA. *p* < 0.05 was considered to be statistically significant.

## Results

### Metabolic parameters in four groups

The mean mice blood glucose level of the NC at days 0, 7, 14, and 28 were 6.87 mmol/l, 7.3 mmol/l, 7.9 mmol/l, and 7.2 mmol/l, respectively, and that of the HF group were 7.5 mmol/l, 7.4 mmol/l, 6.8 mmol/l, and 7.8 mmol/l, respectively. While the blood glucose levels of the HG group were 26.3 mmol/l, 24.5 mmol/l, 25.0 mmol/l, and 24.9 mmol/l, and that of the HFHG group were 21.7 mmol/l, 24.5 mmol/l, 26.0 mmol/l, and 26.3 mmol/l. The blood glucose level in the HG and HFHG groups were stable and above 16.9 mmol/l (Fig. 1c).

Blood lipid detection showed that plasma concentrations of TC, TG, LDL-C and HDL-C in HF and HFHG group at days 0, 7, 14, and 28 were higher than those in NC group, respectively (*p* < 0.05). On the other hand, HG group showed no significant difference in blood lipid levels compared with NC group (*p* > 0.05) (Fig. 1d).

### The bone regeneration in four groups

**Fig. 2:**
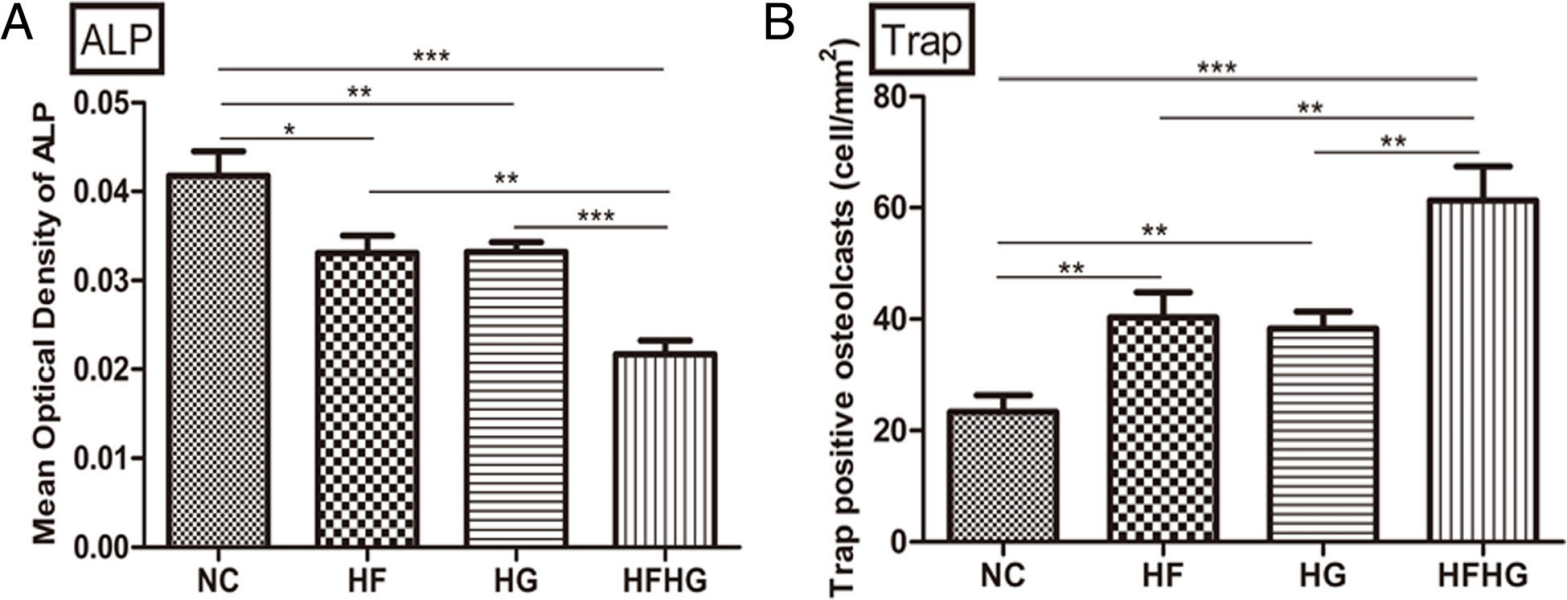
The bone regeneration in the four groups on day 7 after operation. **a** The expression of ALP was the lowest in HFHG group. **b** The number of TRAP-positive osteoclasts was the highest in HFHG group. *p < 0.05, **p < 0.01, ***p < 0.001

On day 7, HE and Masson staining showed the newly formed bone matrix at the edges in NC group (Additional file 1: Figure S1A, S1B). IHC showed that ALP expression was positive at the edge of the bone defect area in the four groups, and the expression was the lowest in HFHG group (*p* < 0.05, Additional file 1: Figure S1C, Fig. 2a). Compared with the control group, the number of TRAP-positive osteoclasts in the other groups was significantly increased and was the highest in HFHG group (*p* < 0.05, Additional file 1: Figure S1D, Fig. 2b).

**Fig. 3:**
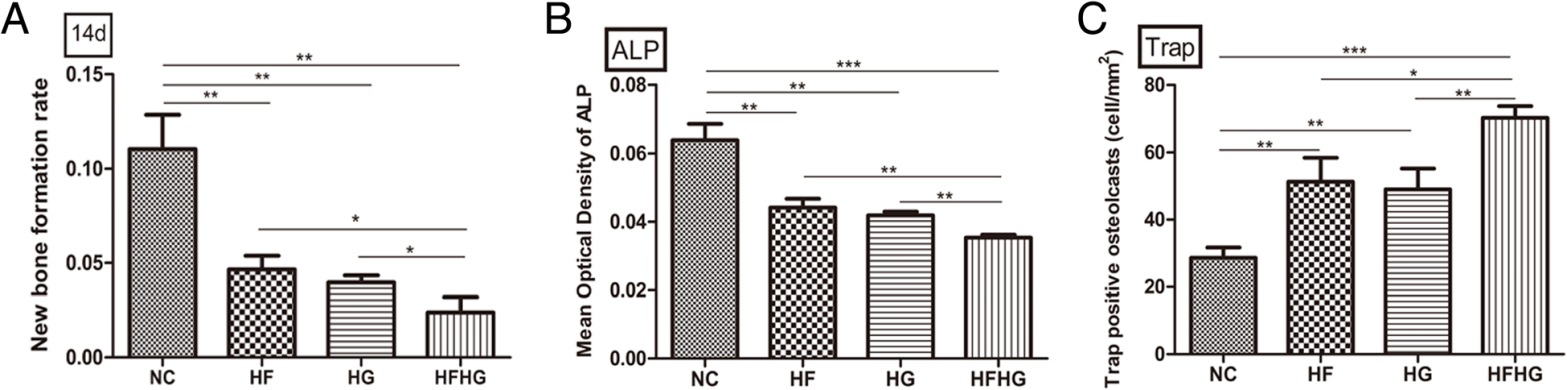
The bone regeneration in the four groups on day 14 after operation. **a** The new bone fraction rate was the lowest in HFHG group. **b** The expression of ALP in NC group was significantly higher compared to the other three groups and was the lowest in HFHG group. **c** The number of TRAP-positive osteoclasts was the highest in HFHG group. *p < 0.05, **p < 0.01, ***p < 0.001

On day 14, HE and Masson staining showed the newly formed bone tissue at the edge of the defect area and the new bone had gradually maturing intersections in the NC group bone. The new bone fraction rate in HFHG group was significantly different from the other three groups (*p* < 0.05, Additional file 2: Figure S2A, S2B, Fig. 3a). The expression of ALP in HFHG group was significantly different from the other three groups (*p* < 0.05, Additional file 2: Figure S2C, Fig. 3b). Moreover, the number of TRAP-positive osteoclasts was the highest in HFHG group (*p* < 0.05, Additional file 2: Figure S2D, Fig. 3c).

**Fig. 4:**
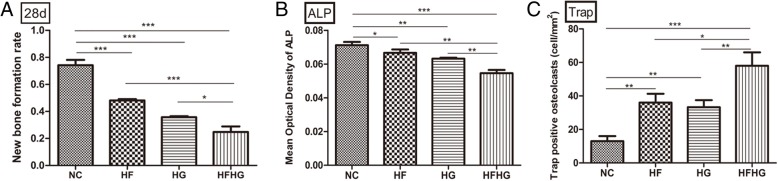
The bone regeneration in the four groups on day 28 after operation. **a** The new bone area was smaller in HF, HG, and HFHG groups compared to NC group. **b** The expression of ALP was weaker in HFHG group compared to the other three groups. **c** The number of TRAP-positive osteoclasts was the highest in HFHG group. *p < 0.05, **p < 0.01, ***p < 0.001

On day 28, the defect area of NC group was filled with new bone tissue. The defect area of HF, HG, and HFHG groups was occupied by a new bone and fibrous tissue, and the new bone area and the maturity of the bone in HFHG group were the lowest among the four groups (Additional file 3: Figure S3a, b, Fig. 4a). The expression level of ALP in HFHG group was also the lowest among the four groups (*p* < 0.05, Additional file 3: Figure S3C, Fig. 4b). The number of TRAP-positive osteoclasts in HFHG group was higher than in three other groups (*p* < 0.05, Additional file 3: Figure S3D, Fig. 4c).

### The expression of Runx2 and TAZ in four groups

**Fig. 5:**
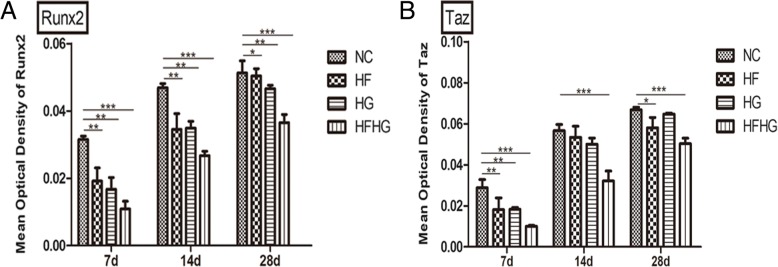
IHC staining of Runx2 and TAZ in the four groups. **a** Compared with NC group, the expression of Runx2 was weaker in the other three groups and was the lowest in HFHG group. **b** The expression of TAZ showed a significant difference between NC and HFHG groups, but not in HF and HG groups. *p < 0.05, **p < 0.01, ***p < 0.001

**Fig. 6:**
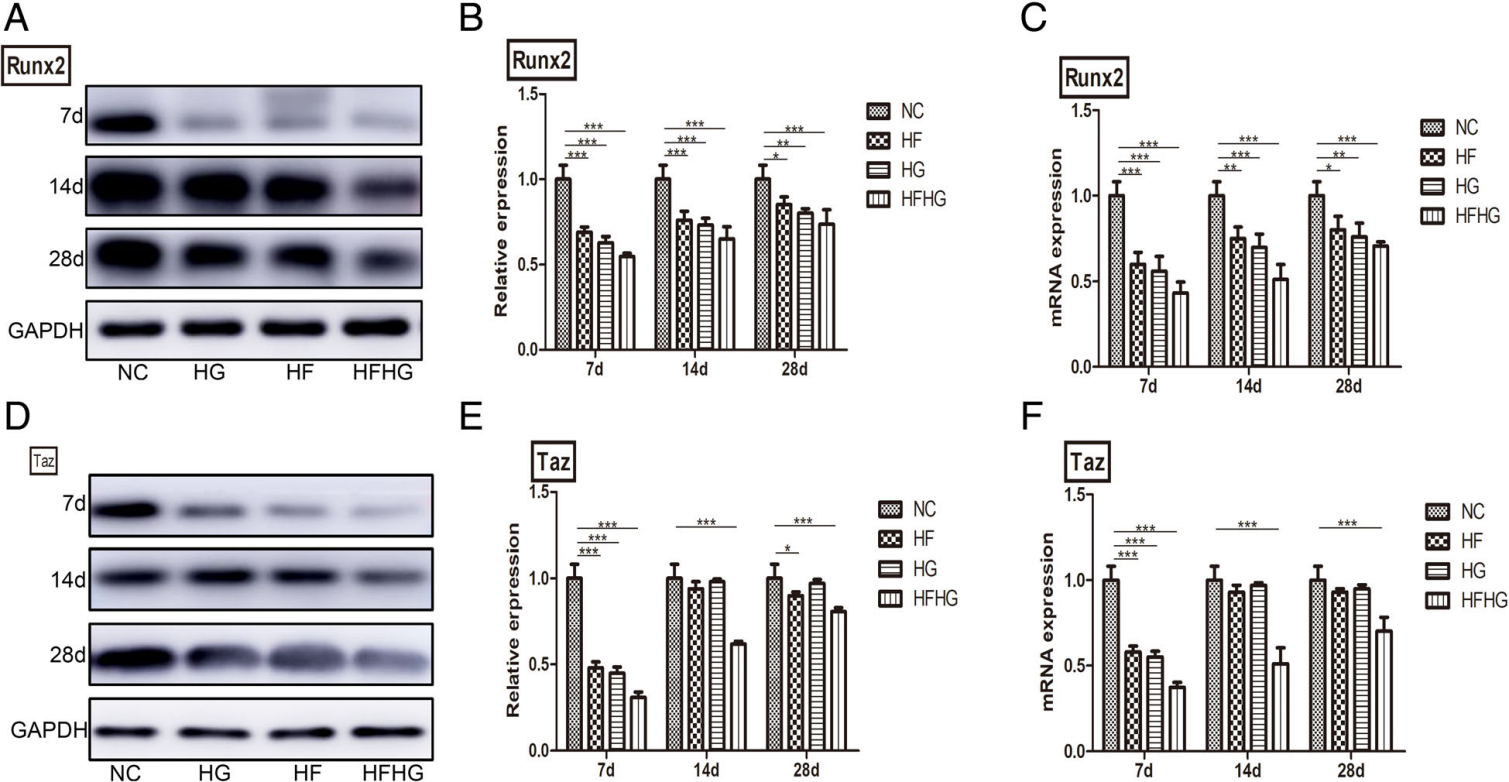
RT-PCR and Western blot analysis of Runx2 and TAZ expression in the four groups. **a–c** The expression of Runx2 in HFHG group was the lowest. **d–f**, The expression of TAZ was weaker in HFHG group than in NC group, but showed no significant difference between HF and HG groups. *p < 0.05, **p < 0.01, ***p < 0.001

IHC showed that Runx2 and TAZ expression was positive in granulation tissue as inflammatory granulation tissue appeared and was positive in vascular endothelial cells and fibroblast-like cells on day 7. With the bone healing, the new bone tissue appeared in the defect area. Then, the trabecular bone was arranged loosely in the new bone tissue (Additional file 4: Figure S4A–S4C, S4D–S4F). IHC showed positive expression of Runx2 and TAZ in osteoblasts around new bone tissue. Compared with the control group, the expression of Runx2 was weaker in the other three groups during bone regeneration and was the lowest in HFHG group (*p* < 0.05, Figs. 5a and 6a–c). The expression of TAZ in HG group was significantly different from NC group only at day 7, and only slightly decreased at day 14 and 28 with no statistical difference. The expression of TAZ in HG group also showed a slight decrease at day 14 compared with the normal group, with no statistical difference, but the expression of TAZ in HG group showed a significant decrease at day 7 and 28 (*p* < 0.05, Figs. 5b and 6d–f).

## Discussion

To investigate the process of bone regeneration under a high-fat and high-glucose microenvironment, we established mice model. In HFHG group, the mice showed the characteristics of hyperglycemia and hyperlipidemia. Thus, high-fat and high-glucose mice model was successful and could be used to simulate a high-fat and high-glucose microenvironment in patients with hyperlipidemia and T2DM.

Next, bone regeneration in the four groups was detected by HE staining, Masson staining, IHC of ALP, and TRAP staining, the results showed the following: (1) compared with NC group, bone regeneration was inhibited in HF, HG, and HFHF groups; (2) compared with other three groups, the inhibition of bone regeneration was the highest in HFHG group.

Two main reasons may explain the link between T2DM and hyperlipidemia: the metabolic factors such as insulin resistance and abdominal obesity [28] and the lack of insulin and insulin resistance leading to defects in the removal of LDL-C and TG [29]. Consistent with previous results that T2DM patients had mixed lipid disorders, including elevated levels of TG and LDL-C [30, 31], this study showed that LDL-C and TG levels were significantly higher in HFHG group compared to HF group. Previous studies have shown that the oxidation products of LDL-C regulate bone metabolism by acting on osteoblasts [32, 33]. In addition, LDC-C and oxidized LDL can stimulate p53-mediated apoptosis of osteoblasts [15]. Furthermore, in vitro culture of mice bone marrow mesenchymal stem cells confirmed that LDL-C oxides stimulated their transformation into adipocytes [34]. In this study, high LDL-C level in HFHG group may reduce the number and differentiation rate of osteoblasts while increase the number of osteoclasts, thereby decreasing the rate of new bone formation. Consistent with the findings of the postmenopausal women not taking hormone replacement therapy that TG and LDL levels were negatively associated with bone mineral density (BMD) in all measured sites [35], we found that the area of the mature new bone in HFHG group was the lowest among the four groups. Another study was to investigate the associations of nutrient patterns (NP) with BMD and fractures in the US adults. The results showed a healthy nutrient-dense NP, characterized by high intakes of minerals, vitamins and fiber, benefit BMD independent of potential confounding factors. In contrast, adherence to a high-energy NP characterized by high consumption of total and saturated fats, carbohydrates. and sugar pose a risk for low BMD. [36].

Bone tissue regeneration is a dynamic process regulated by the balance between bone formation and bone resorption. Under physiological conditions, the formation and resorption of the bone is a delicate and tightly regulated process [37]. The differentiation of osteoblasts is a key factor in the process of bone formation. Runx2 and TAZ play an important role in osteoblast formation and function [38, 39]. In this study, we investigated the expression of Runx2 and TAZ in the four groups. Compared with NC group, the expression of Runx2 was weaker in the other three groups and was the lowest in HFHG group. These observations are consistent with our data that the inhibition of bone regeneration was the highest in HFHG group. However, the expression of TAZ was only significantly decreased in HFHG group, but not in HF and HG groups, compared to NC group. In high-glucose condition, the expression of TAZ did not appear to be significantly reduced [40]. Therefore, in high-fat and high-glucose environment the expression of TAZ showed the same trend as Runx2 due to their interaction. TAZ-Runx2 is a key pathway to promote MSC osteogenesis [41]. In addition, Phorbaketal A stimulated osteoblast differentiation through TAZ-mediated Runx2 activation, which led to bone formation [24]. Therefore, we speculate that the inhibition of bone regeneration under a high-fat and high-glucose microenvironment is related to the reduced expression of Runx2 and TAZ, but further mechanistic studies are needed to confirm it.

## Conclusion

Our findings suggest that bone regeneration is significantly inhibited under a high-fat and high-glucose microenvironment compared to a simple high-fat condition or high-glucose condition. Runx2 and TAZ are involved in the inhibition of bone regeneration under the high-fat and high-glucose microenvironment, but the precise mechanism needs further investigations.

## Additional files

Additional file 1: **Figure S1.** The bone regeneration in the four groups on day 7 after operation. A–B HE staining (A) and Masson staining (B) of the defect area showed the newly formed bone matrix at the edges in NC group. Scale, 100 μm. C The expression of ALP was the weakest in HFHG group. Scales, 20 μm. D the number of TRAP-positive osteoclasts was the highest in HFHG group. Scales, 50 μm. (TIF 91889 kb)

Additional file 2: **Figure S2.** The bone regeneration in the four groups on day 14 after operation. A HE staining of the defect area showed that the new bone tissue appeared at the edge of the defect area in NC group. Scale, 100 μm. B Masson staining of the defect area showed that the newly formed bone in NC group had immature bone and mature bone intersections. Scale, 100 μm. C The expression of ALP in NC group was significantly higher compared to the other three groups, and was the lowest in HFHG group. Scale, 20 μm. D The number of TRAP-positive osteoclasts was the highest in HFHG group. Scale, 50 μm. (TIF 90473 kb)

Additional file 3: **Figure S3.** The bone regeneration in the four groups on day 28 after operation. A The newly formed bone tissue filled the defect area in NC group. Scale, 100 μm. B The proportion of mature bone was high in NC group. A mature bone was formed in HF, HG, and HFHG groups. Scale, 100 μm. C The expression of ALP was weaker in HFHG group compared to the other three groups. Scale, 20 μm. D The number of TRAP-positive osteoclasts was the highest in HFHG group. Scale, 50 μm. (TIF 84670 kb)

Additional file 4: **Figure S4.** IHC staining of Runx2 and TAZ in the four groups. A–C Compared with NC group, the expression of Runx2 was weaker in the other three groups and was the lowest in HFHG group. Scale, 20 μm. D–F The expression of TAZ showed a significant difference between NC and HFHG groups, but not in HF and HG groups. Scale, 20 μm. (TIF 59170 kb)

## Abbreviations

BMD: Bone mineral density
BMP2: Bone morphogenetic protein 2
DDW: Double distilled water
HDL-C: High-density lipoprotein
IHC: Immunohistochemistry
LDL-C: Low-density lipoprotein
NP: Nutrient patterns
OSX: Osterix
Runx2: Runt-related transcription factor 2
STZ: Streptozotocin
T2DM: Type 2 diabetes mellitus
TAZ: Transcriptional co-activator with PDZ-binding domain
TC: Total cholesterol
TG: Triglycerides
