# Supplementary figures and images for: High-fat and high-glucose microenvironment decreases Runx2 and TAZ expression and inhibits bone regeneration in the mouse
Source: J Orthop Surg Res. 2019 Feb 18;14:55. doi: 10.1186/s13018-019-1084-2 (PMC6380030; doi:10.1186/s13018-019-1084-2)

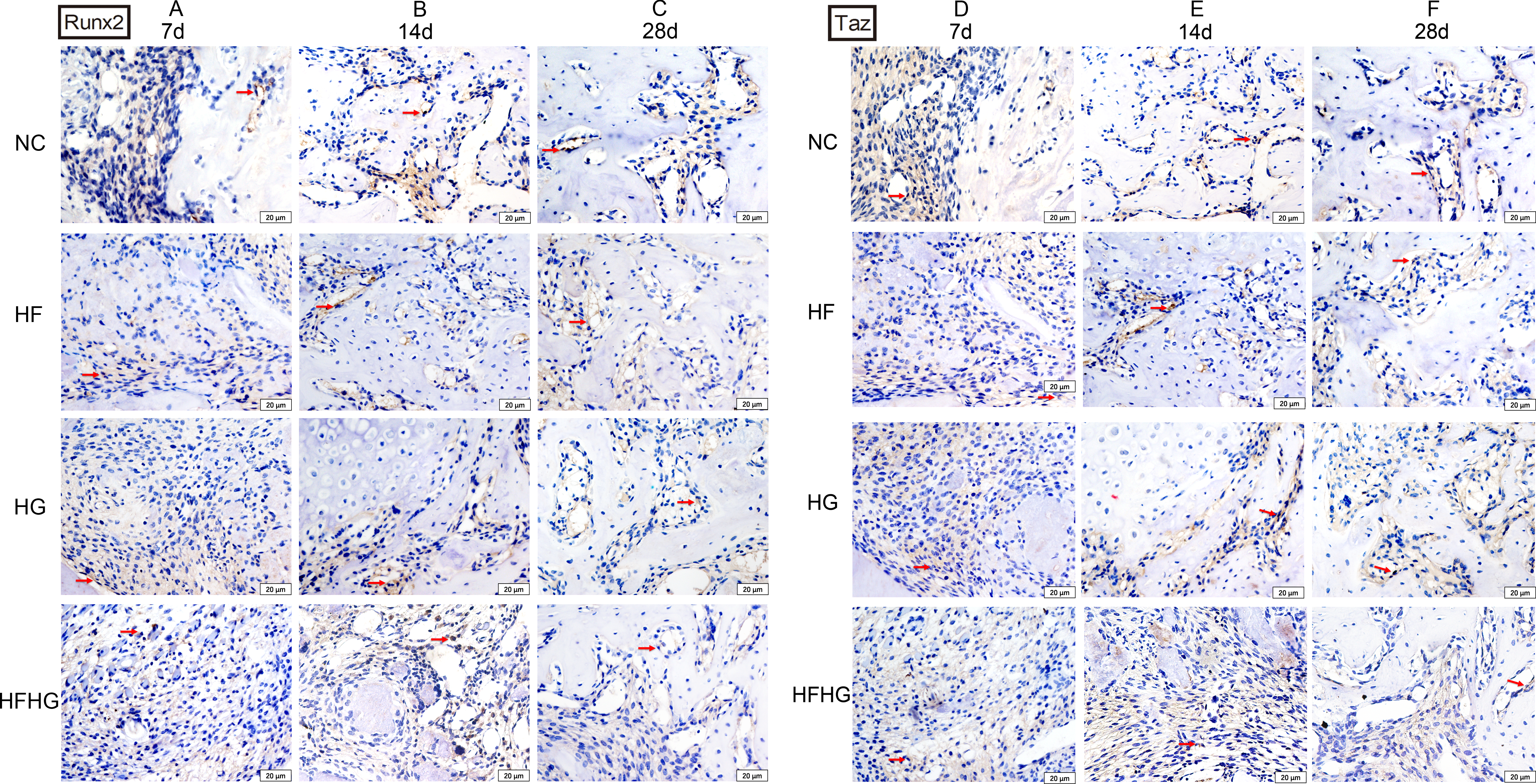

Supplement: Supplementary file 4 — Figure S4. IHC staining of Runx2 and TAZ in the four groups. A–C Compared with NC group, the expression of Runx2 was weaker in the other three groups and was the lowest in HFHG group. Scale, 20 μm. D–F The expression of TAZ showed a significant difference between NC and HFHG groups, but not in HF and HG groups. Scale, 20 μm. (TIF 59170 kb) [file 13018_2019_1084_MOESM4_ESM.tif]
